# Enhancement of Anti-Inflammatory Activity of Curcumin Using Phosphatidylserine-Containing Nanoparticles in Cultured Macrophages

**DOI:** 10.3390/ijms17060969

**Published:** 2016-06-20

**Authors:** Ji Wang, Yu-Xia Kang, Wen Pan, Wan Lei, Bin Feng, Xiao-Juan Wang

**Affiliations:** State Key Laboratory of Military Stomatology & National Clinical Research Center for Oral Diseases & Shaanxi Engineering Research Center for Dental Materials and Advanced Manufacture, Department of Pharmacy, School of Stomatology, The Fourth Military Medical University, 145 Changle West Road, Xi’an 710032, China; wjsydkq145@163.com (J.W.); kangyuxia1030@163.com (Y.-X.K.); 15691087927@163.com (W.P.); 15129627980@163.com (W.L.); bingelfeng@hotmail.com (B.F.)

**Keywords:** phosphatidylserine, curcumin, nanostructured lipid carriers, anti-inflammatory, macrophages

## Abstract

Macrophages are one kind of innate immune cells, and produce a variety of inflammatory cytokines in response to various stimuli, such as oxidized low density lipoprotein found in the pathogenesis of atherosclerosis. In this study, the effect of phosphatidylserine on anti-inflammatory activity of curcumin-loaded nanostructured lipid carriers was investigated using macrophage cultures. Different amounts of phosphatidylserine were used in the preparation of curcumin nanoparticles, their physicochemical properties and biocompatibilities were then compared. Cellular uptake of the nanoparticles was investigated using a confocal laser scanning microscope and flow cytometry analysis in order to determine the optimal phosphatidylserine concentration. *In vitro* anti-inflammatory activities were evaluated in macrophages to test whether curcumin and phosphatidylserine have interactive effects on macrophage lipid uptake behavior and anti-inflammatory responses. Here, we showed that macrophage uptake of phosphatidylserine-containing nanostructured lipid carriers increased with increasing amount of phosphatidylserine in the range of 0%–8%, and decreased when the phosphatidylserine molar ratio reached over 12%. curcumin-loaded nanostructured lipid carriers significantly inhibited lipid accumulation and pro-inflammatory factor production in cultured macrophages, and evidently promoted release of anti-inflammatory cytokines, when compared with curcumin or phosphatidylserine alone. These results suggest that the delivery system using PS-based nanoparticles has great potential for efficient delivery of drugs such as curcumin, specifically targeting macrophages and modulation of their anti-inflammatory functions.

## 1. Introduction

Macrophages play a crucial role in the development and progression of atherosclerosis (AS) [1]. During AS progression, one of the effector cells in atherosclerotic plaques is a macrophage that can actively ingest modified lipids such as oxidized low density lipoprotein (oxLDL), resulting in the formation of foam cells, a hallmark of atherosclerotic plaque [2]. These foam cells can then secrete a panel of pro-inflammatory cytokines, including tumor necrosis factor (TNF), interleukin 6 (IL-6) and monocyte chemoattractant protein 1 (MCP-1), which together amplify inflammatory responses [3]. Therefore, activation of anti-inflammatory functions of macrophages is of particular interest. Also, development of delivery carriers containing anti-inflammatory drugs that can specifically target macrophages intracellularly may have great potential for the treatment of AS.

Recently, lipid-based nanoparticles were tested as a drug delivery system of anti-inflammatory drugs in the suppression of TNF-α production in macrophages [4,5]. To improve the specificity of this drug delivery system targeting the macrophages, one of the attractive routes is to include the anionic lipid phosphatidylserine (PS) in the drug delivery systems. PS is normally present in the inner leaflet of the cell plasma membranes, and is retained there by the enzyme aminophospholipid translocase [6]. During the early stage of cell apoptosis, PS is translocated to the outer leaflet of the cell plasma membrane, and the exposure of PS provides an “eat-me” signal to initiate phagocytosis of apoptotic cells by phagocytic cells, such as macrophages [7,8,9,10]. Evidence in the literature shows that many types of nanomaterials have harnessed this particular mechanism of PS for either regulating immunological functions in different models of inflammation [11,12,13,14,15] or directing drugs and contrast agents to the sites enriched with phagocytic cells [16,17,18,19]. For example, it was previously reported that liposomes with PS presenting in the lipid bilayer could increase angiogenesis that promoted infarct repair and downregulated inflammation via secretion of anti-inflammatory factors, such as interleukin 10 (IL-10) and transforming growth factor β (TGF-β) [19]. Another common use of PS exists mainly in medical imaging, such as magnetic resonance imaging (MRI), fluorescence, ultrasound, and single photon emission computed tomography (CT). With PS-based modification, carriers containing imaging probes are able to target inflammatory sites where innate immune cells such as macrophages and dendritic cells play a primary role [20,21,22,23]. However, the effect of PS-coated carriers on the activity of an anti-inflammatory drug has seldom been reported.

Curcumin, a natural polyphenolic compound extracted from the rhizome of turmeric (*Curcuma longa*), is well known for its anti-inflammatory, anti-oxidative, and cholesterol metabolism regulating properties [24,25,26]. However, its clinical application has been greatly limited due to its low solubility, poor bioavailability, and degradation at alkaline pH. Many studies have tried to develop effective carriers for curcumin in order to improve its solubility and stability [27,28,29]. Some of these formulations have additional effects for cellular delivery or intracellular release of curcumin [30,31].

**Figure 1:**
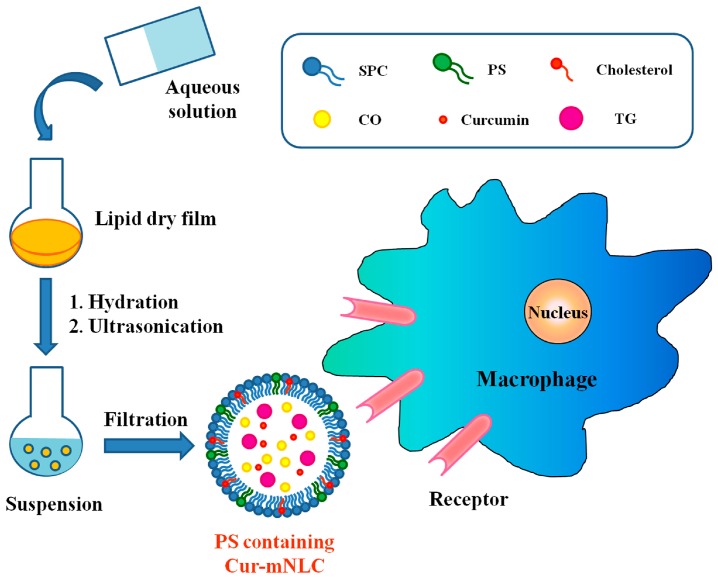
A schematic diagram of preparation procedures of PS-based nanoparticles and of endocytotic uptake by RAW 264.7 macrophages.

Herein, based on the specific targeting properties and anti-inflammatory inducing effects of PS-containing carriers on macrophages, PS-containing nanostructured lipid carriers (mNLCs) were designed to incorporate curcumin into PS-containing nanoparticles. We hypothesized that combination of PS-containing carriers and curcumin may achieve drug intracellular delivery as well as enhance drug anti-inflammatory activities. The objective of this study was to examine NLCs’ uptake and the consequent functional changes in cultured macrophages. The main scheme of our present study is presented in Figure 1.

## 2. Results

### 2.1. Particle Size, Zeta Potential, Entrapment Efficiency (EE), Drug Loading Capacity (DL), and Morphology

The average sizes, polydispersity index (PDI), zeta potentials, EE and DL of different PS-containing curcumin-loaded nanostructured lipid carriers (Cur-mNLCs) are all listed in Table 1. The average sizes of different PS-containing Cur-mNLCs were all around 200 nm, which could be preferentially taken up by macrophages in the aorta [32]. Meanwhile, zeta potentials of Cur-mNLCs increased as the added amount of PS increased, indicating negatively charged PS had been successfully inserted into the phospholipid monolayer. Furthermore, when PS mol % reached 8% and beyond, zeta potentials became constant, suggesting the saturation of PS in the phospholipid layer [18]. The EE and DL of various preparations were not significantly changed with the increment of PS amount (4%–12%). However, when the PS amount reached 20%, the EE and DL decreased remarkably. Hence, only 0%–12% of PS molar ratios was used in the following studies.

**Figure 2:**
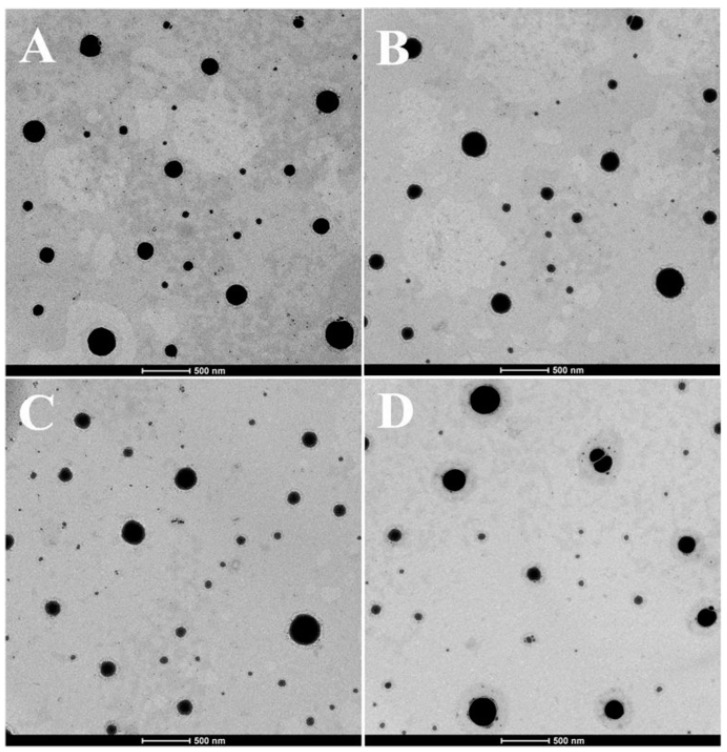
Transmission electron microscopy (TEM) images of different PS-containing Cur-mNLCs. (**A**) 0% PS; (**B**) 4% PS; (**C**) 8% PS; (**D**) 12% PS.

As shown in Figure 2, different containing Cur-mNLCs all exhibited typical spherical structures (presented as black spherical spots).

### 2.2. In Vitro Drug Release Studies

**Figure 3:**
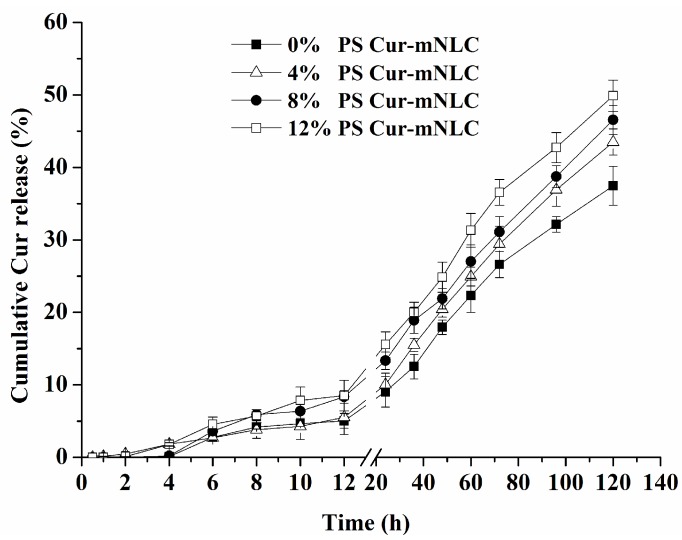
*In vitro* release profiles of different PS-containing Cur-mNLCs (0%, 4%, 8% and 12% PS molar ratios) in 200 mL of modified phosphate buffered saline (PBS) buffer solution (pH 7.4, containing 0.5% sodium dodecyl sulfate (SDS)) at 37 °C for 120 h (mean ± SD, *n* = 3). The cumulative drug release of Cur-mNLCs was all below 50% at 120 h. All preparations displayed a similar release manner without significant differences (*p* > 0.05).

Figure 3 illustrates *in vitro* drug release behavior of different PS-containing Cur-mNLCs. The release rate of curcumin among the Cur-mNLCs with different amounts of PS was not statistically different, indicated by the fact that the average of curcumin release rate per 20 h during the period of 40 h to 120 h of incubation was 6.7% in Cur-mNLCs with 0% PS, 7.4% with 4% PS, 7% with 8% PS, and 7.5% with 12% PS (*p* > 0.05). The percentages of accumulative release of curcumin entrapped in Cur-mNLCs were all below 50% at 120 h, indicating sustained release of curcumin in these preparations.

### 2.3. Hemocompatibility Assay

No obvious hemolytic effects were observed in all Cur-mNLCs (Table 2) with hemolytic activities of all formulations below 5%, which confirmed that Cur-mNLCs possessed excellent blood compatibility [33].

### 2.4. Cytotoxicity Studies

**Figure 4:**
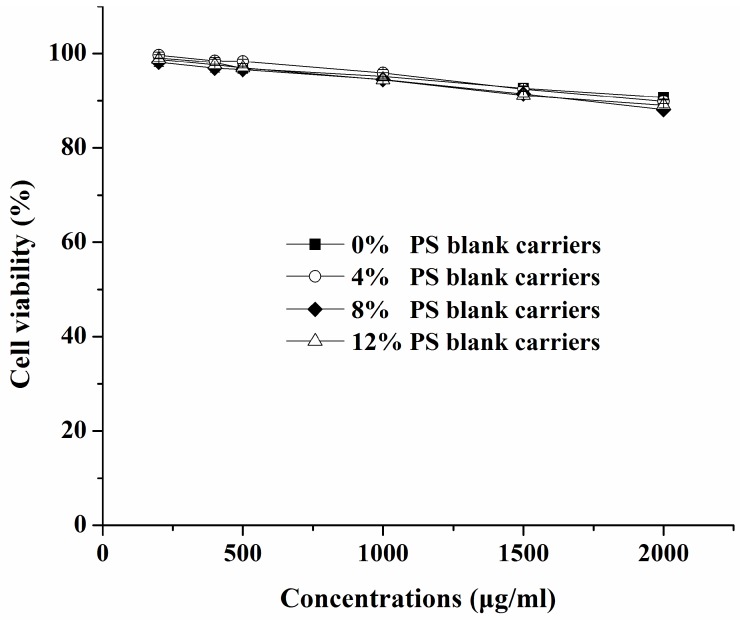
*In vitro* cytotoxicities of blank carriers with different PS amounts (0%, 4%, 8%, and 12%) at concentrations ranging from 200 to 2000 μg/mL. Blank carriers were non-toxic in the concentrations of 200–500 μg/mL, and the cells’ viability was decreased as the carriers’ concentrations increased (mean ± SD, *n* = 3). All preparations showed negligible cytotoxicities without significant differences between groups (*p* > 0.05).

**Figure 5:**
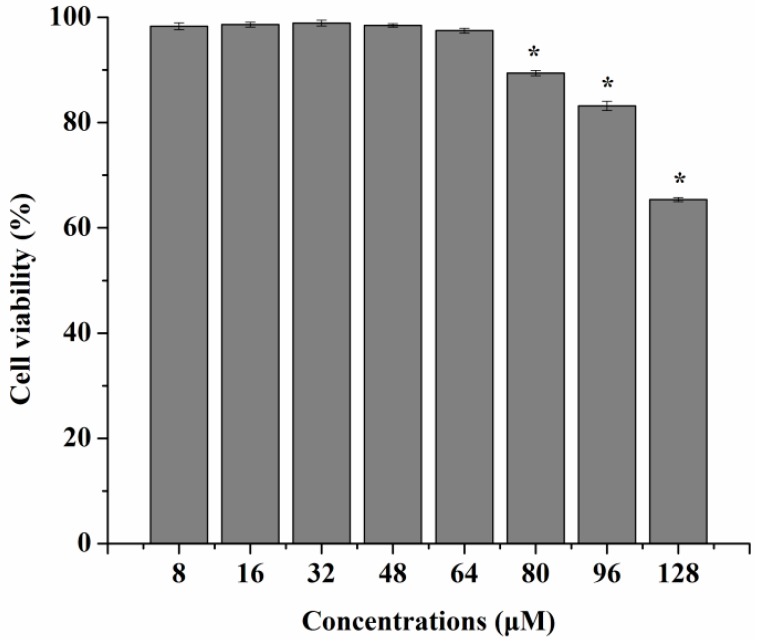
*In vitro* cytotoxicities of curcumin solutions at different concentrations ranging from 8 to 128 μM. When concentrations of curcumin solutions were in the range of 8–64 μM, the cell viability was not affected at all (mean ± SD, *n* = 3) (* *p* < 0.05, compared with curcumin solutions at 64 μM).

**Figure 6:**
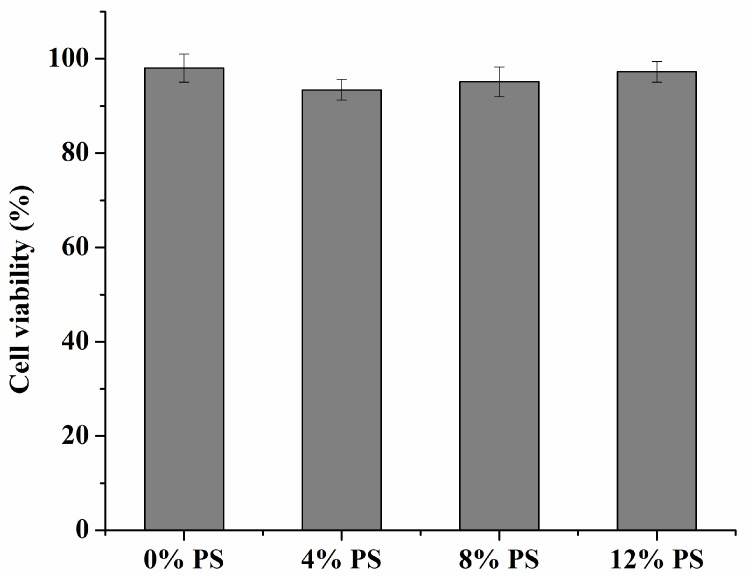
*In vitro* cytotoxicities of PS-containing Cur-mNLCs at the same concentrations as used in the cellular uptake and anti-inflammatory cell studies, with equal concentrations of curcumin (20 μM) and carriers (expressed as solid content, about 303 μg/mL). No significant cytotoxicity against RAW 264.7 cells was found in any Cur-mNLCs (mean ± SD, *n* = 3) (*p* > 0.05).

Figure 4 shows that all the blank carriers with different amounts of PS slightly reduced cell viability only at the higher concentrations (>500 μg/mL) of the carrier, which was not statistically different among these groups (*p* > 0.05). The slight decrease in cell viability might result from the higher concentrations of lipids or the hyperosmolarity of the medium. As seen in Figure 5, when concentrations of curcumin solutions were in the range of 8–64 μM, the cells’ viability was not affected at all (*p* < 0.05). Meanwhile, no significant cytotoxicity against RAW 264.7 cells was found in any Cur-mNLCs (Figure 6). All the results demonstrated that the concentrations of the preparations (with equal concentrations of curcumin at 20 μM and carriers expressed as solid content at 303 μg/mL) used in the subsequent studies were safe to macrophages.

### 2.5. In Vitro Cellular Uptake

**Figure 7:**
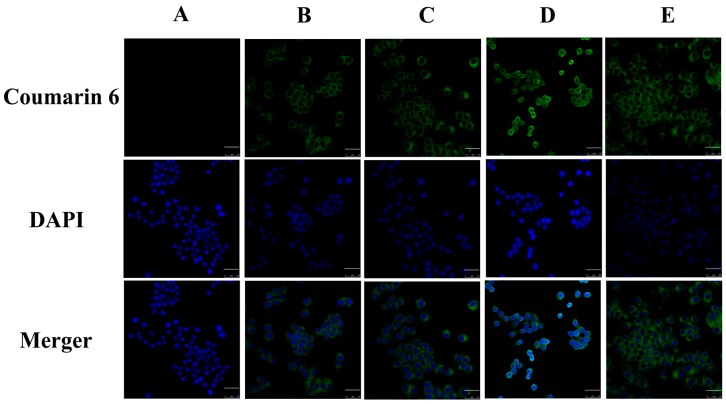
CLSM images. (**A**) negative control group (blank culture medium without NLCs); (**B**) 0% PS containing C6-mNLCs; (**C**) 4% PS containing C6-mNLCs; (**D**) 8% PS containing C6-mNLCs; (**E**) 12% PS containing C6-mNLCs. The cellular uptake of C6-mNLCs was increased with an increasing amount of PS in the nanoparticles, up to 8% PS. Also, the uptake was decreased when PS concentrations reached 12%. Scale bar is 25 µm.

**Figure 8:**
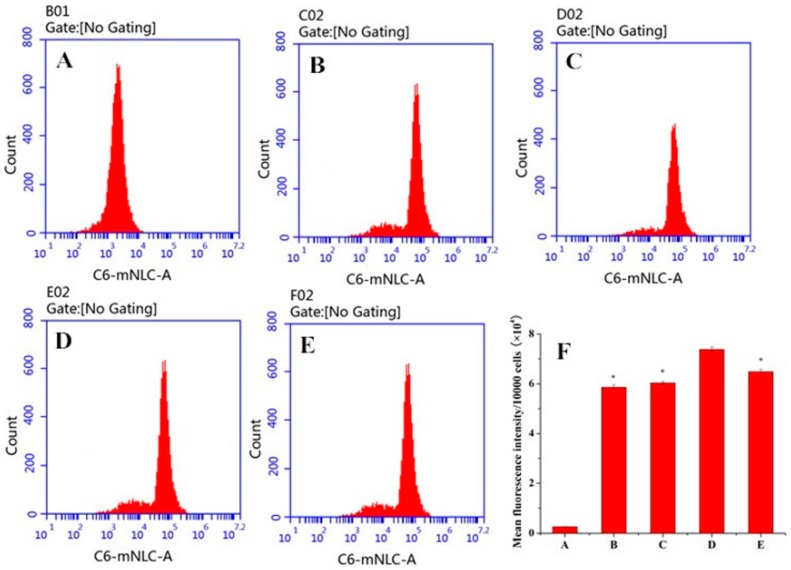
Flow cytometry analysis. (**A**) negative control group (blank culture medium without NLCs); (**B**) 0% PS containing C6-mNLCs; (**C**) 4% PS containing C6-mNLCs; (**D**) 8% PS containing C6-mNLCs; (**E**) 12% PS containing C6-mNLCs; (**F**) mean fluorescence density analysis; Significant difference, * *p <* 0.05, compared with D. In panel F, the letters of A–E in the X axis correspond to Group A–E, respectively. In accordance with CLSM results, the cellular uptake of C6-mNLCs was increased with an increasing amount of PS in the nanoparticles, up to 8% PS. Also, the uptake was decreased when PS concentrations reached 12% (mean ± SD, *n* = 6).

Figure 7 presents confocal laser scanning microscopy (CLSM) images of macrophages after 3 h incubation with different PS-containing coumarin 6 (C6)-mNLCs at 100 ng/mL of C6 concentration. The images were obtained from C6 channel (green), 4′,6-diamidino-2-phenylindole (DAPI) channel (blue) and merger of the two channels. All C6-mNLCs (green) were densely dispersed around the blue nuclei (stained by DAPI), indicating the internalization of fluorescent nanoparticles into macrophages. Moreover, both fluorescent microscopy (Figure 7) and flow cytometry analysis (Figure 8) revealed that the cellular uptake of C6-mNLCs was increased with an increasing amount of PS in the nanoparticles, up to 8% PS. Also, the uptake was decreased when PS concentrations reached 12%. Consequently, 8% PS containing mNLCs were chosen as optimized preparations for subsequent studies (*p <* 0.05, mean fluorescent density of D compared with that of either B, C or E) (Figure 8F).

### 2.6. Enhanced Anti-Inflammatory Functions of Curcumin by PS in Cultured Macrophages 

#### 2.6.1. Lipid Uptake Behavior of Macrophages

**Figure 9:**
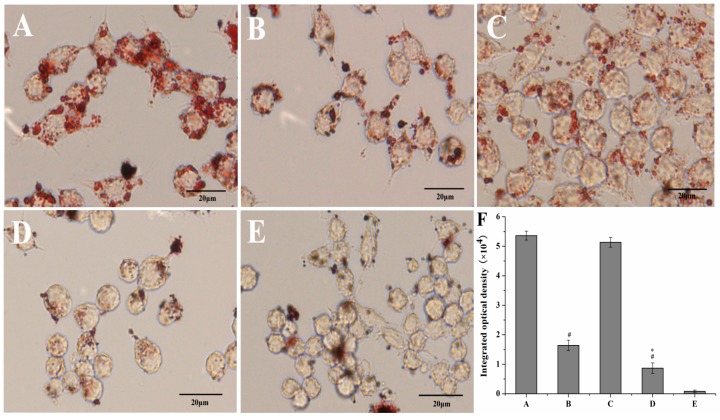
Oil Red O staining and integrated optical density of oxLDL-induced macrophages. (**A**) positive control group; (**B**) curcumin solutions; (**C**) blank PS-containing carriers; (**D**) Cur-mNLCs; (**E**) negative control group; (**F**) integrated optical density analysis; # *p <* 0.01, compared with A, * *p <* 0.05, compared with B. In panel F, the letters of A–E in the X axis correspond to Group A–E, respectively. Curcumin solutions or Cur-mNLCs markedly reduced oxLDL-induced cholesterol accumulation in macrophages (*p <* 0.01 for Group B or D *vs*. Group A), and Cur-mNLCs exhibited more potent effects than curcumin solutions (*p <* 0.05 for Group D *vs*. Group B). PS did not have an obvious effect on macrophages’ lipid uptake behavior (*p* > 0.05 for Group C *vs*. Group A) (mean ± SD, *n* = 6). Scale bar is 20 µm.

**Figure 10:**
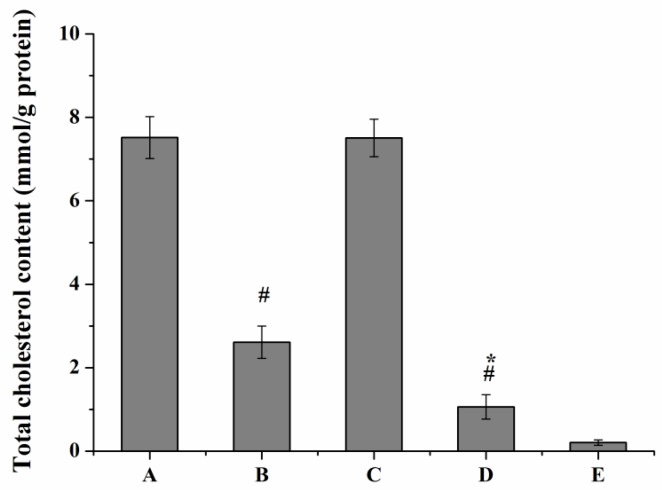
Total cholesterol content of oxLDL-induced macrophages. (A) positive control group; (B) curcumin solutions; (C) blank PS-containing carriers; (D) Cur-mNLCs; (E) negative control group; # *p <* 0.01, compared with A, * *p <* 0.05, compared with B. In accordance with Oil Red O staining results, curcumin solutions or Cur-mNLCs markedly reduced oxLDL-induced cholesterol accumulation in macrophages (*p <* 0.01 for Group B or D *vs*. Group A), and Cur-mNLCs exhibited more potent effects than curcumin solutions (*p <* 0.05 for Group D *vs*. Group B). PS did not have an obvious effect on macrophages’ lipid uptake behavior (*p* > 0.05 for Group C *vs*. Group A) (mean ± SD, *n* = 6).

Oil Red O staining and intracellular cholesterol measurement was performed to investigate whether curcumin and PS had interacting effects on macrophage lipid uptake behavior. Treatment of curcumin solutions or Cur-mNLCs markedly reduced oxLDL-induced cholesterol accumulation in macrophages (*p <* 0.01 for Group B or D *vs*. Group A), and Cur-mNLCs exhibited more potent effects than curcumin solutions (*p <* 0.05 for Group D *vs.* Group B), both verified by Oil Red O staining and cellular cholesterol content (Figure 9 and Figure 10). While, PS did not have an obvious effect on macrophage lipid uptake behavior, as indicated by the insignificant *p* values of integrated optical density of Oil Red O and total cellular cholesterol content between blank PS-containing carriers and positive control groups (*p* > 0.05 for Group C *vs.* Group A).

#### 2.6.2. Inflammatory Responses of Macrophages

**Figure 11:**
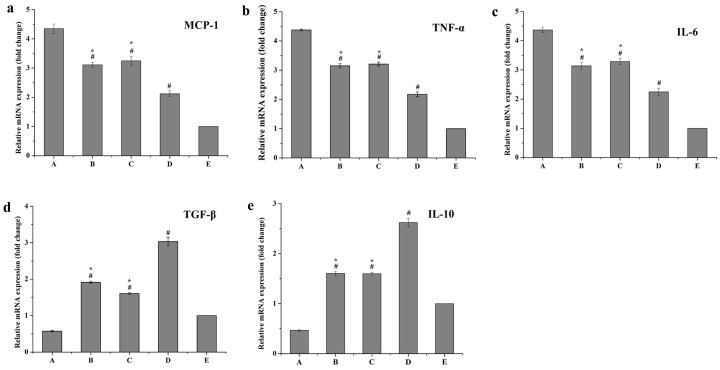
mRNA expression levels of pro-inflammatory factors (MCP-1, TNF-α, IL-6) and anti-inflammatory factors (TGF-β and IL-10). (A) positive control group; (B) curcumin solutions; (C) blank PS-containing carriers; (D) Cur-mNLCs; (E) negative control group; # *p <* 0.01, compared with A, * *p <* 0.01, compared with D; (**a**) RT-qPCR assay for mRNA levels of MCP-1; (**b**) RT-qPCR assay for mRNA levels of TNF-α; (**c**) RT-qPCR assay for mRNA levels of IL-6; (**d**) RT-qPCR assay for mRNA levels of TGF-β and (**e**) RT-qPCR assay for mRNA levels of IL-10 (mean ± SD, *n* = 6). mRNA expression levels (fold change) of all pro-inflammatory cytokines were remarkably reduced in the curcumin solutions group (B), blank PS-containing carriers group (C) and Cur-mNLCs group (D), compared with the positive control group (A) (*p <* 0.01 for Group B, C, or D *vs.* Group A). The combination of curcumin and PS (Cur-mNLCs) exerted a more potent inhibiting effect, in comparison with curcumin or PS alone (*p <* 0.01 for Group B or C *vs*. Group D). Curcumin solutions (Group B), blank PS-containing carriers (Group C) and Cur-mNLCs (Group D) all enhanced the mRNA expression levels of anti-inflammatory cytokines, compared with the positive control group (*p <* 0.01 for Group B, C, or D *vs.* group A). Among the three treatment groups (Groups B, C, and D), Group D displayed the most significant effects (*p <* 0.01 for Group B or C *vs*. Group D).

**Figure 12:**
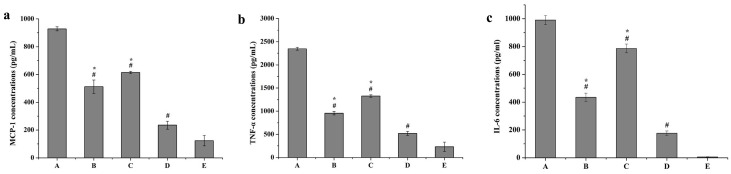

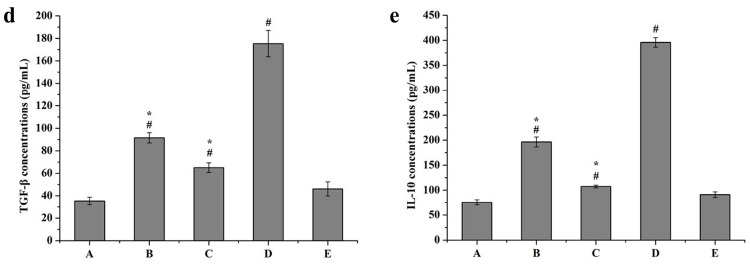
Protein expression levels of pro-inflammatory factors (MCP-1, TNF-α, IL-6) and anti-inflammatory factors (TGF-β and IL-10). (A) positive control group; (B) curcumin solutions; (C) blank PS-containing carriers; (D) Cur-mNLCs; (E) negative control group; # *p <* 0.01, compared with A, * *p <* 0.01, compared with D; (**a**) ELISA assay for expression of MCP-1; (**b**) ELISA assay for expression of TNF-α (**c**) ELISA assay for expression of IL-6; (**d**) ELISA assay for expression of TGF-β and (**e**) ELISA assay for expression of IL-10 (mean ± SD, *n* = 6). Protein expression levels (fold change) of all pro-inflammatory cytokines were remarkably reduced in the curcumin solutions group (B), blank PS-containing carriers group (C) and Cur-mNLCs group (D), compared with the positive control group (A) (*p <* 0.01 for Group B, C, or D *vs*. Group A). The combination of curcumin and PS (Cur-mNLCs) exerted a more potent inhibiting effect, in comparison with curcumin or PS alone (*p <* 0.01 for Group B or C *vs*. Group D). Curcumin solutions (Group B), blank PS-containing carriers (Group C) and Cur-mNLCs (Group D) all enhanced the protein expression levels of anti-inflammatory cytokines, compared with the positive control group (*p <* 0.01 for Group B, C, or D *vs*. Group A). Among the three treatment groups (Groups B, C, and D), Group D displayed the most significant effects (*p <* 0.01 for Group B or C *vs*. Group D).

Figure 11 and Figure 12 show that mRNA expression levels (fold change) and protein expression levels (concentrations) of all pro-inflammatory cytokines were remarkably reduced in the curcumin solutions group (B), blank PS-containing carriers group (C) and Cur-mNLCs group (D), compared with the positive control group (A) (*p <* 0.01 for Group B, C, or D *vs*. Group A). The combination of curcumin and PS (Cur-mNLCs) exerted a more potent effect on inhibiting the production and release of pro-inflammatory factors by macrophages, in comparison with curcumin or PS alone (*p <* 0.01 for Group B or C *vs.* Group D). 

In contrast, curcumin solutions (Group B), blank PS-containing carriers (Group C) and Cur-mNLCs (Group D) all enhanced the mRNA and protein expression levels of anti-inflammatory cytokines, compared with the positive control group (*p <* 0.01 for Group B, C, or D *vs.* Group A). Among the three treatment groups (Groups B, C, and D), Group D displayed the most significant effects on promoting the production and release of anti-inflammatory factors (*p <* 0.01 for Group B or C *vs.* Group D).

## 3. Discussion

A common feature of atherosclerotic plaques is the presence of inflammatory macrophages. These macrophages commonly upregulate the expression of pro-inflammatory cytokines after they are transformed into lipid-laden foam cells, which directly contribute to the progression of AS and other cardiovascular diseases [34]. Importantly, foam cells play a key role in the engulfment of apoptotic cells and oxidized lipids. Therefore, a nanoscale carrier that mimics apoptotic cells and/or oxidatively modified lipids may have an enhanced ability to deliver agents to atherosclerotic plaques.

In the current study, PS was incorporated into a nanostructured lipid carrier (NLC) to aid in targeting inflammatory macrophages as well as act as an anti-inflammatory agent for resolution of inflammation. Besides, the potent anti-inflammatory drug curcumin was loaded in the core of PS-containing NLCs, aiming to deliver more drugs to macrophages and enhance the anti-inflammatory effects of curcumin.

In order to select an optimal PS concentration for successful targeting to macrophages, NLCs with different PS amounts (4%, 8%, 12%, and 20%) were prepared by a thin-film dispersion method. A conventional phosphatidylcholine NLC (0% PS) was also prepared as a negative control. All NLCs had a hydrodynamic diameter of about 200 nm, which may accumulate more in the atherosclerotic lesions as indicated by the results from Chono *et al*. [35]. They reported that aortic biodistribution of liposomes with different sizes (70 nm, 200 nm, and 500 nm) in atherogenic mice was size-dependent, and the aortic concentration of 200 nm liposomes was the highest of all. This could be due to the higher vascular permeability and higher uptake rate by macrophages in the atherosclerotic lesions of 200 nm liposomes when compared with particles with either smaller or bigger sizes (70 nm or 500 nm). The presence of PS in the nanoparticles was confirmed by a gradual change in zeta potential to more negative values. A slight change in zeta potential was observed when the PS molar ratio was over 8%, indicating the PS concentration in the phospholipid layer was saturated [18]. When the PS molar ratio was increased to 20%, EE and DL of curcumin decreased significantly. It may possibly be explained by the fact that overload of PS in the phospholipid monolayer changed the membrane rigidity or stability leading to poor drug incorporation [23]. Thus 0%–12% PS concentrations were subsequently investigated in this study for physicochemical characterizations and macrophage uptake. 

Transmission Electron Microscopy (TEM) images showed that all PS-containing Cur-mNLCs were spherical nanoparticles, with diameters coinciding with dynamic light scattering (DLS) data. The *in vitro* release experiments exhibited less than 50% release after 120 h, indicating these nanoparticles could slowly release curcumin and thus act as highly efficient, controlled drug delivery carriers. No significant hemolytic effects in the hemocompatibility study were observed, which confirmed that Cur-mNLCs possessed excellent blood compatibility (<5% hemolysis) [33]. The *in vitro* cytotoxicity assay revealed that the concentrations of blank PS carriers, curcumin solutions, and PS-containing Cur-mNLCs used in the cellular uptake and anti-inflammatory studies were safe and non-toxic. Additionally, it could be inferred that inhibition of lipid uptake behavior and pro-inflammatory responses of macrophages was not caused by cytotoxic effects of those preparations [25]. 

C6 has been widely used as a fluorescent probe to mark nanoparticles for investigating cellular uptake behavior [36]. In this study, C6 was used instead of curcumin in the nanoparticles to observe and analyze cellular uptake of Cur-mNLCs aimed to select the optimal concentration of PS. CLSM and flow cytometry (FCM) studies demonstrated that macrophages engulfed more fluorescent material as PS concentrations reached up to of 8%, with a decrease in uptake when PS concentrations were over 12%. It was reported that the molar ratio of PS in the membranes of mammalian cells was in the range of 2–10 mol %, and macrophages would preferentially recognize and engulf nanoparticles that expressed approximate physiological concentrations of PS, like 8 mol % PS in this article. Thus, 8% PS concentrations were used in subsequent *in vitro* anti-inflammatory studies [23]. The above results also showed that PS incorporation facilitated intracellular delivery of nanoparticles to macrophages, when compared with conventional phosphatidylcholine NLC (0% PS).

The oxLDL is pro-inflammatory in nature which may activate and initiate an inflammatory process [3]. Lesional macrophages could be activated to produce and release inflammatory cytokines after they ingest modified lipoproteins such as oxLDL [37]. Thus inhibition of oxLDL uptake by macrophages may attenuate cell transformation into the inflammatory state. In this study, oxLDL was applied to initiate the inflammatory responses of macrophages and oxLDL uptake (indicated by the cellular cholesterol content) was evaluated by Oil Red O staining and intracellular cholesterol determination [26]. Then the effect of PS incorporation on curcumin-mediated oxLDL uptake behavior and anti-inflammatory responses was studied in cultured macrophages. Oil Red O staining and intracellular cholesterol measurement demonstrated that curcumin alone or Cur-mNLCs could significantly inhibit lipid uptake behavior of macrophages (*p <* 0.01, compared with the positive control group), while PS alone had little effect on cholesterol accumulation (*p* > 0.05, compared with the positive control group). Cur-mNLCs exhibited a more significant influence on the lipid uptake in comparison with curcumin alone (*p <* 0.05), which may be due to their better intracellular delivering properties mediated by PS on their shells [38]. 

PS nanoparticles have been explored in a number of systemic and local inflammation models as anti-inflammatory and immunomodulatory agents [39,40]. In the present study, both mRNA and protein expression levels of inflammatory cytokines were determined to evaluate whether PS could enhance anti-inflammatory responses in macrophages mediated by curcumin. Figure 11 and Figure 12 show significantly greater transcription and secretion levels of anti-inflammatory factors (TGF-β and IL-10) from oxLDL activated macrophages after the uptake of PS-containing Cur-mNLCs, compared with curcumin or PS alone (*p <* 0.01). In contrast, lower levels of pro-inflammatory cytokines (MCP-1, TNF-α, and IL-6) were found following the uptake of PS-containing Cur-mNLCs, in comparison with curcumin or PS alone (*p <* 0.01). These findings suggest that combination of curcumin and PS may exert a more potent effect on modulating anti-inflammatory functions of macrophages, similar to those results reported previously [41,42]. As PS-containing Cur-mNLCs may act as a signal for engulfment by macrophages, they are recognized by these cells specifically, resulting in promoted drug cellular delivery [43]. Besides, rapid phagocytosis of PS-containing Cur-mNLCs may have a direct effect on anti-inflammatory cytokine production, similar to downstream recognition of the dead cells [44,45]. Finally, these two properties of PS contribute to the excellent performance of PS-containing Cur-mNLCs on resolving inflammation of macrophages.

## 4. Materials and Methods

### 4.1. Materials

Curcumin (purity of 98%) was purchased from Aladdin Industrial Incorporation (Ontario, CA, USA); soybean phosphatidylcholine (SPC, Lipoid S 100) from Lipoid GmbH (Ludwigshafen, Germany); PS (1,2-diacyl-sn-glycero-3-phospho-L-serine), cholesterol, C6, Oil Red O and 3-(4,5-dimethyl-thiazol-2-yl)-2,5-diphenyltetrazolium bromide (MTT) from Sigma-Aldrich (St. Louis, MO, USA); cholesteryl oleate (CO) from Alfa Aescar/Thermo Fisher Scientific (Lancashire, UK); trioleate glycerol (TG) from Tokyo Chemical Industry Co., Ltd. (Tokyo, Japan), and oxLDL from Yiyuan Biotech. Co., Ltd. (Guangzhou, Guangdong, China). For cell culture, Dulbecco’s modified Eagle’s medium (DMEM) with glutamate, fetal bovine serum (FBS) and antibiotic solution (penicillin/streptomycin, 0.1% *v*/*v*) were all from Sigma-Aldrich. HPLC grade reagents were used as mobile phase in HPLC determination, and all other chemicals and solvents were of analytical grade. Deionized water (18.2 MΩ) was supplied with an analytic ultra-pure water system (ELGA, High Wycombe, UK).

Male Sprague-Dawley rats (7–9 weeks old; body weight: 200 ± 20 g) were purchased from the Experimental Animal Research Center of the Fourth Military Medical University (Xi’an, Shaanxi, China) and used for *in vitro* hemocompatibility assay. Whole blood samples were collected from the orbit. All studies were approved and supervised by the Animal Care and Use Committee of the Fourth Military Medical University according to the Chinese Council on Animal Care guidelines.

### 4.2. Preparation of Different PS-Containing Cur-mNLCs

Different PS-containing Cur-mNLCs were prepared by a thin-film dispersion method [46]. Briefly, solutions of a lipid mixture and curcumin (molar ratios as indicated in Table 3) in chloroform were mixed and the solvent was evaporated in vacuum. The prepared dry film was then dispersed with 15 mL of PBS buffer (0.05 M, pH 6.5) for 30 min. Then the mixture was vortexed thoroughly for 5 min, and further ultrasonicated for 6 min with an Ultra-Homogenizer (Scientz-IID, Ningbo, China) at 300 W to obtain a uniform suspension. The product was finally extruded through a 0.22-μm filter several times.

### 4.3. Particle Size and Zeta Potential

Droplet size, PDI, and zeta potential of different PS-containing Cur-mNLCs were determined at 25 °C by DLS with a Nano-ZS90 (Malvern, UK) Prior to measurement, 100 μL of each sample was diluted with 10 mL deionized water. Measurements were carried out in triplicate on three independent preparations.

### 4.4. Entrapment Efficiency and Drug Loading Capacity

The EE and DL of different PS-containing Cur-mNLCs (three batches each) were calculated after removing the untrapped drug with a mini-column centrifugation method [47]. The amount of drug incorporated in the Cur-mNLCs (m) and the total amount of drug (m_0_) were assayed by HPLC at 426 nm, respectively. The HPLC system was composed of a Diamonsil (5 μm, 250 mm × 4.6 mm) column, a UV detector (Shimadzu, Japan) operated at 426 nm and a pump (LC-2010A). The mobile phase was acetonitrile: 4% acetic acid (48:52, *v*/*v*); the flow rate was maintained at 1.0 mL/min; the column temperature was set at 30 °C.

The EE for curcumin was calculated with the equation below:

EE(%) = m/m_0_ × 100 (1)

While the DL for curcumin was calculated as follows:

DL(%) = m/(m + m_1_) × 100 (2)

where m_1_ stands for the weight of other lipids in mNLCs.

### 4.5. Transmission Electron Microscopy

The morphology of different PS-containing Cur-mNLCs was examined by TEM (Tecnai G2, FEI, Hillsboro, TX, USA). Each sample was diluted appropriately for observation.

### 4.6. In Vitro Drug Release Studies of Various PS-Containing Cur-mNLCs

A dialysis bag method was used to investigate the *in vitro* drug release behavior of Cur-mNLCs. Three milliliters of Cur-mNLC dispersions were loaded into a dialysis bag (8000–14,000 Da) and suspended in 200 mL of modified PBS buffer solution (pH 7.4, containing 0.5% SDS) at 37 ± 0.5 °C. Samples (1 mL) were collected at different time points for a period of 120 h and subjected to HPLC assay as described above. All *in vitro* drug release studies were performed in triplicate.

### 4.7. Hemocompatibility Assay

The degree of hemolysis induced by Cur-mNLCs on rat blood was determined. Fresh blood samples from male Sprague-Dawley rats were centrifuged at 1500 rpm for 15 min, washed four times with normal saline and suspended in the same buffer at the concentration of 2%. Subsequently, 2.5 mL of erythrocyte suspension were added to 2.2 mL of normal saline and 0.3 mL of different Cur-mNLCs (three batches each), incubated at 37 °C for 3 h and then centrifuged for 10 min at 3000 rpm. Blood was similarly incubated with normal saline or deionized water as negative or positive control, respectively. The amount of hemoglobin released into the supernatant was determined at 576 nm. The percentage of HR was calculated by the formula:

HR(%) = (At − An)/(Ap − An) × 100
(3)

where At, An, Ap are the absorbance of test samples, negative samples and positive samples, respectively. 

### 4.8. Macrophage Culture

RAW 264.7 macrophages were supplied by American Type Cell Culture (ATCC, Manassas, VA, USA). Cells were grown in DMEM supplemented with 10% FBS, penicillin (100 U/mL) and streptomycin (100 mg/mL), and incubated in a 5% CO_2_ humidified incubator at 37 °C.

### 4.9. Cytotoxicity Assay

RAW 264.7 cells were seeded at 5 × 10^4^ cells/well in 96-well plates and cultured for 24 h. After removing the medium, cells were then incubated with 200 μL fresh medium containing different PS blank mNLCs controls—curcumin null (concentrations 200~2000 μg/mL), curcumin solutions (8~128 μM curcumin) or PS-containing Cur-mNLCs (20 μM curcumin) for 24 h, respectively. 

After this, 20 μL of MTT (5 mg/mL in pH 7.4 PBS) was added and the plates were incubated for 4 h under the same condition. Finally, the supernatant was discarded and 150 μL of dimethyl sulfoxide was added to dissolve the formed MTT formazan crystals. The absorbance was recorded at 490 nm by an ELISA plate reader (Thermo Scientific, Waltham, MA, USA). All experiments were performed in triplicate.

The concentration of drug carriers (generally defined as the solid content) was calculated by the following equation [48]:

Solid content (mg/mL) = Total weight of lipid materials/Volume of the preparation
(4)

### 4.10. In Vitro Cellular Uptake

To facilitate the observation of cellular uptake, a fluorescent agent C6 was loaded into mNLCs instead of the drug. Different PS-containing C6-loaded mNLCs (C6-mNLCs) were prepared in the same way as Cur-mNLCs, except that curcumin was substituted by the fluorescent marker. The unencapsulated C6 was removed through the dialysis method.

For confocal microscopy analysis, RAW 264.7 cells were seeded on glass-bottom dishes and incubated for 24 h. Different C6-mNLCs (containing 100 ng/mL C6) were added and incubated for 3 h at 37 °C. Then cells were washed with PBS three times, fixed with 4% paraformaldehyde (*v*/*v*) for 20 min, followed by cell nuclei staining with DAPI for 10 min. Finally, cells were examined by a confocal laser scanning microscope (CLSM, Leica, DMI3000B, Wetzlar, Germany).

For flow cytometry analysis, RAW 264.7 cells were seeded into 6-well plates and cultured for 24 h. Cells were treated with different C6-mNLCs (containing 100 ng/mL C6). After incubation for 3 h, the cells were washed, trypsinized, harvested, and re-suspended in PBS. The intensity of fluorescence inside the cell was detected by FCM (BD-C6, Franklin Lakes, NJ, USA).

According to the above qualitative and quantitative assays, the PS-containing NLCs with highest cellular uptake were used in the subsequent studies.

### 4.11. Enhanced Anti-Inflammatory Functions of Curcumin by PS in Cultured Macrophages

Curcumin, blank PS-containing carrier and Cur-mNLC were incubated with oxLDL and macrophages respectively, in order to investigate the effects of drug and carrier on modulating oxLDL uptake behavior and anti-inflammatory responses of macrophages. 

Specifically, RAW 264.7 were seeded at a density of 1 × 10^5^ cells/well in 12-well plates, and grown at 37 °C and 5% CO_2_ in fully humidified air for 24 h. The medium was discarded and cells were washed with PBS three times. Then cells were incubated with fresh serum-free DMEM, containing 50 μg/mL oxLDL and different preparations respectively. The experimental groups were divided into three groups: Group B (curcumin solutions with curcumin concentration of 20 μM); Group C (blank PS-containing carriers, with PS concentration equal to that of Cur-mNLCs); Group D (Cur-mNLCs, with curcumin concentration of 20 μM). Besides, cells incubated with fresh serum-free DMEM only containing 50 μg/mL oxLDL were taken as positive control group (denoted as Group A), and cells incubated with fresh serum-free DMEM only were taken as negative control group (denoted as Group E). After being cultured further for a period of time, all groups were investigated for the following items, respectively.

#### 4.11.1. Lipid Uptake Behavior of Macrophages

##### Oil Red O Staining

After 24 h incubation, cells were washed three times with PBS, fixed with 10% paraformaldehyde for 30 min and then stained with 0.5% Oil Red O. Cells were observed by microscope (IX 71, Olympus, Japan) at ×400 magnifications, respectively. Meanwhile, the integrated optical density was also quantified by Image-Pro Plus 6 as an indicator of cellular lipid content density.

##### Intracellular Cholesterol Measurement

After 24 h incubation, cells were washed three times with PBS. Intracellular cholesterol was extracted by a mixture of hexane/isopropanol (3:2, *v*/*v*). The supernatant was dried under nitrogen flush after cellular debris was removed. The total cholesterol content in cells was assayed by the commercial cholesterol quantification kit (Sigma-Aldrich).

#### 4.11.2. Anti-Inflammatory Responses of Macrophages

##### Real-Time Quantitative PCR (RT-qPCR) Assay for mRNA Levels of Inflammatory Factors

mRNA expressions of inflammatory factors in each group were detected by RT-qPCR. Total RNA was isolated from macrophages with Trizol reagent (Invitrogen, Waltham, MA, USA) according to the manufacturer’s instructions. cDNAs were synthesized by using a first strand synthesis kit (Invitrogen, San Diego, CA, USA). Aliquots of the cDNAs were then amplified with specific primers for inflammatory cytokines, respectively. Glyceraldehyde 3-phosphate dehydrogenase (GAPDH) primers were used as an internal control. The primer sequences of MCP-1, TNF-α, IL-6, TGF-β, IL-10 and GAPDH were as follows: MCP-1 sense, 5′-GCTTCTGGGCCTGTTGTTCA-3′ and antisense, 5′-TCTTGTAGCCCTCCAGCCTA-3′; TNF-α sense, 5′-GAAAGCATGATCCGCGACGT-3′ and antisense, 5′-CGAAGTTCAGTAGACAGAAG-3′; IL-6 sense, 5′-AGTTGCCTTCTTGGGACTGA-3′, antisense, 5′-CCACGATTTCCCAGAGAACA-3′; TGF-β sense, 5′-AGACATTCGGGAAGCAGTGC-3′, antisense, 5′-AAAGACAGCCACTCAGGCGT-3′; IL-10 sense, 5′-ACTGCTATGCTGCCTGCTCT-3′, antisense, 5′-TTCACCTGGCTGAAGGCAGT-3′; GAPDH sense, 5′-CCGAGAATGGGAAGCTTGTC-3′, antisense, 5′-AAGCACCAACAGAGGAGAA-3′. The RT-qPCR conditions were: 94 °C for 3 min followed by 40 cycles (94 °C for 20 s, 55 °C for 20 s, and 72 °C for 20 s). All relative expressions of genes were normalized to GAPDH. 

##### Detection of Inflammatory Cytokines in Cell Culture Medium by ELISA

After 24 h incubation, supernatants in cell cultures were collected and centrifuged at 12,000 rpm for 10 min. The production of inflammatory cytokines in the supernatant was determined by ELISA (Biosource, Camarillo, CA, USA) according to the manufacturer’s protocol.

### 4.12. Statistical Analysis

Data were presented as mean ± SD. One-way ANOVA was performed for comparing the differences between experimental groups. Also, the differences were considered as significant when *p* < 0.05.

## 5. Conclusions

The results from this study demonstrated the potential of using PS-coated nanoparticles to deliver an anti-inflammatory drug for efficient targeting of macrophages and activating their anti-inflammatory responses. The combination of curcumin and PS in the nanoparticles exhibited superior cellular drug delivery and anti-inflammatory effects than curcumin or PS alone, suggesting that this product may be promising for treating those diseases, including AS, where macrophage inflammation has pathophysiological relevance. However, there remains further investigation in *in vivo* models.

## Figures and Tables

**Table 1:** Particle size, PDI, Zeta potential, EE and DL of different PS containing Cur-mNLCs (mean ± standard deviation (SD), *n* = 3).

Samples	Particle Size (nm)	PDI	Zeta Potential (mV)	EE (%)	DL (%)
0% PS	212.1 ± 1.41	0.076 ± 0.002	−1.33 ± 0.41	85.64 ± 0.67	4.04 ± 0.05
4% PS	210.6 ± 1.27	0.060 ± 0.003	−18.01 ± 0.54	86.13 ± 0.56	3.64 ± 0.03
8% PS	207.6 ± 1.69	0.044 ± 0.005	−45.31 ± 0.36	88.38 ± 0.34	3.96 ± 0.06
12% PS	198.4 ± 1.74	0.047 ± 0.003	−45.56 ± 0.41	88.78 ± 0.58	4.00 ± 0.04
20% PS	156.8 ± 1.25	0.102 ± 0.006	−45.85 ± 0.26	49.69 ± 0.33 *	1.87 ± 0.08 *

* *p <* 0.01, compared with 12% PS Cur-mNLCs.

**Table 2:** Hemolysis assay of different PS-containing Cur-mNLCs (mean ± SD, *n* = 3).

Samples	0% PS	4% PS	8% PS	12% PS
Hemolysis rate (HR) (%)	4.66 ± 0.96	3.36 ± 1.03	3.61 ± 0.87	4.23 ± 0.78

**Table 3:** Preparation of different PS-containing Cur-mNLCs.

Preparation	SPC (mol %)	PS (mol %)	CO (mol %)	TG (mol %)	Cholesterol (mol %)	Curcumin (mol %)
0% PS	45.39	0	12	24	10.46	8.15
4% PS	41.39	4	12	24	10.46	8.15
8% PS	37.39	8	12	24	10.46	8.15
12% PS	33.39	12	12	24	10.46	8.15
20% PS	25.39	20	12	24	10.46	8.15

## Abbreviations

AS	Atherosclerosis
C6	Coumarin-6
C6-mNLCs	PS-containing C6-loaded nanostructured lipid carriers
CLSM	Confocal laser scanning microscope
CO	Cholesteryl oleate
CT	Computed tomography
Cur-mNLCs	PS-containing curcumin-loaded nanostructured lipid carriers
DAPI	4',6-diamidino-2-phenylindole
DL	Drug loading capacity
DLS	Dynamic light scattering
DMEM	Dulbecco’s modified Eagles’s medium
EE	Entrapment efficiency
FBS	Fetal bovine serum
FCM	Flow cytometry
GAPDH	Glyceraldehyde 3-phosphate dehydrogenase
HR	Hemolysis rate
IL-6	Interleukin 6
IL-10	Interleukin 10
MCP-1	Monocyte chemoattractant protein 1
MRI	Magnetic resonance imaging
MTT	3-(4,5-dimethyl-thiazol-2-yl)-2,5-diphenyltetrazolium bromide
NLCs	Nanostructured lipid carriers
oxLDL	Oxidized low density lipoprotein
PBS	Phosphate buffer saline
PDI	Polydispersity index
PS	Phosphatidylserine
RT-qPCR	Real-time quantitative PCR
SDS	Sodium dodecyl sulfate
SPC	Soybean phosphatidylcholine
TEM	Transmission electron microscopy
TG	Trioleate glycerol
TGF-β	Transforming growth factor β
TNF	Tumor necrosis factor
